# Nutritional Implications of Trade-Offs Between Fresh and Processed Potato Products in the United Kingdom (UK)

**DOI:** 10.3389/fnut.2020.614176

**Published:** 2021-01-11

**Authors:** Wisdom Dogbe, Cesar Revoredo-Giha

**Affiliations:** ^1^The Rowett Institute, University of Aberdeen, Aberdeen, United Kingdom; ^2^Rural Economy, Environment and Society, Scotland's Rural College (SRUC), Edinburgh, United Kingdom

**Keywords:** potatoes, EASI demand model, nutrition, Great Britain, scan data

## Abstract

The UK ranks eleven among world potato producing countries with annual per capita production of about 102 kg. Since 2007, the price of potatoes has increased by 44 per cent and UK households have shown a decreasing trend on their purchases of potatoes. At the same time, retailers and manufacturers have been introducing processed potato products, which also has affected the demand for fresh potatoes. This has shifted demand from fresh potatoes to processed potatoes suggesting that consumers substitute fresh potatoes for processed ones. However, the extent to which this affect individual weekly nutritional composition is unknown. The objective of this study is to estimate the nutritional trade-offs between fresh and processed potatoes consumed in the UK using home scanner panel dataset for Great Britain in 2018. Price and expenditure elasticities were estimated using the linearized version of the Exact Affine Stone Index (EASI) Demand System. Using estimated elasticities, we analyzed the implications of substituting fresh potatoes for processed potatoes on nutrient intake. The results, in terms of the degree of substitution between fresh potatoes and processed potato products, suggest that consumers consider new potatoes baby and baking potatoes as substitutes for mashed potatoes. Maris piper potatoes and new potatoes baby are substitutes for frozen chips and other potatoes whilst white old potatoes and other vegetables and salads are complements to frozen chips and other potatoes. Finally, price reductions in the processed potatoes will increase average weekly caloric intake as well as the intakes of saturated fat and sodium. The latter has implications for public health as they are the major causes of cardiovascular diseases and certain cancers.

## Background

Potatoes have been used for food for over 10,000 years in South America; it first spread to European countries—arriving to the United Kingdom in the late 1,500s (1). Today, potatoes are grown and consumed in over 160 countries (2) with over 4,000 cultivars (3). In 2017, the total world potato production was estimated at 388.2 million tons (4). The UK ranks number 11 among world potato producing countries with annual per capita production of about 102 kg.

Potatoes are an important source of many nutrients, including carbohydrates, vitamin C and some B-vitamins (5). For instance, in the mid-1900s, most of the vitamin C in the British diet was obtained from eating potatoes. Today, this has been substituted with 27 per cent of vitamin C intake coming from drinks (including fruit juice), 22 per cent from vegetables and 19 per cent from fruits (6).

**Figure 1:**
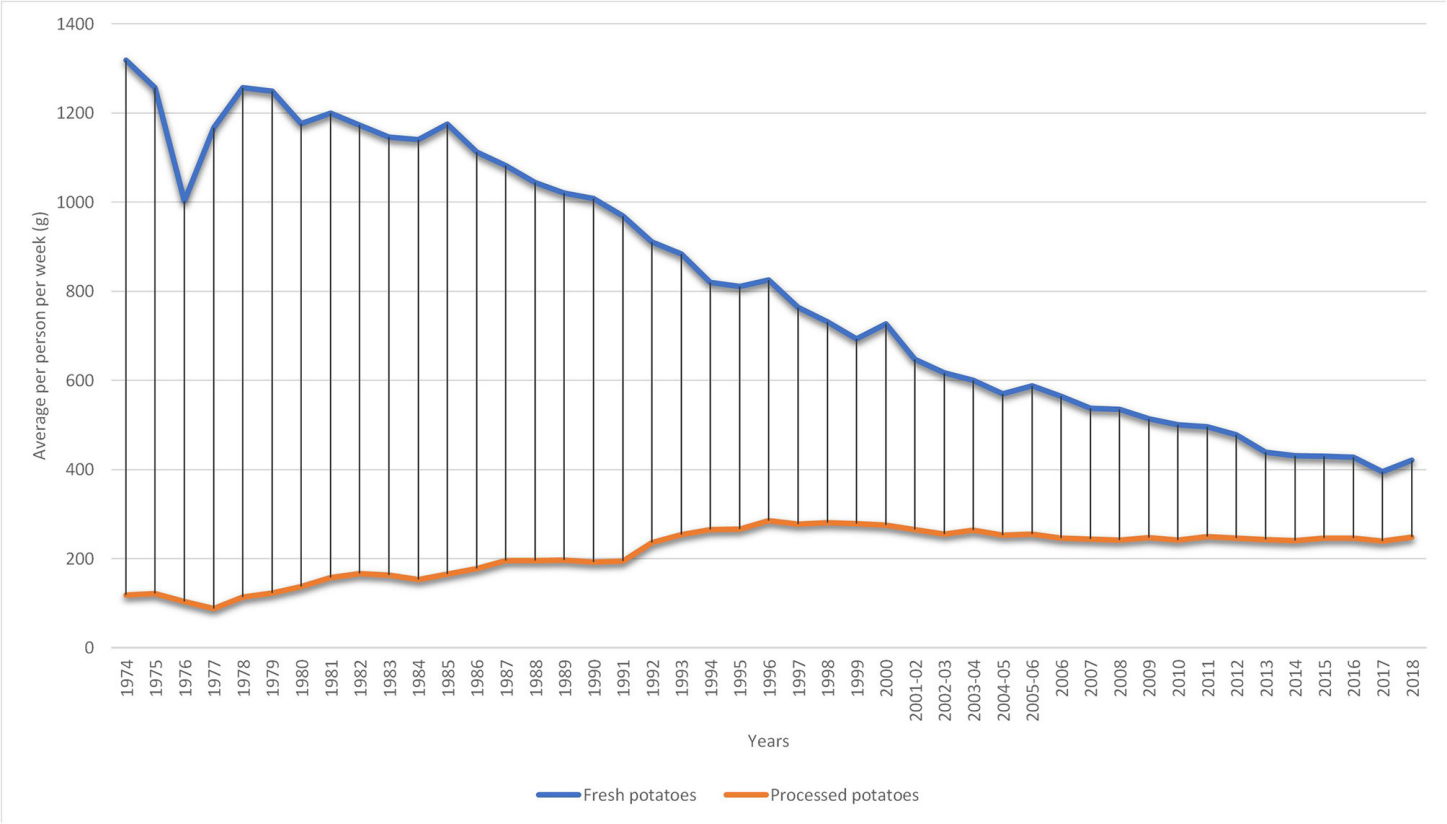
Average weekly consumption of fresh and processed potatoes in the UK. Source: Own elaboration based on DEFRA's data (7).

Potatoes are still a staple of the British diet, with more than 80 per cent of people eating them regularly (5). However, the average weekly consumption of fresh potatoes has declined by 68 per cent from 1974 to 2018. During the same period, the consumption of processed potatoes has increased by 109 per cent (7). Using the DEFRA Family Food dataset, per capita per week purchase of both processed (including chips and takeaway chips, instant potato, canned potatoes, crisps, and potato snacks and other processed potato products, frozen, or not frozen) and fresh potatoes from 1974 to 2018 are shown in Figure 1.

Figure 1 shows that the average total purchase of potatoes (both fresh and processed) is on the decline at a rate^1^ of 0.02 per cent. However, decomposing the graph into fresh and processed potatoes based on DEFRA dataset show that the weekly per capita purchase of fresh potatoes has seen a downward trend since 1974 at a rate of 0.03 per cent. The purchase of processed potatoes, even though, lower has seen a steady growth (of 0.02 per cent) in purchase from 1974 to 2018. Many factors have been related to the downward trend in the purchase of fresh potatoes. First, there have been reduction in the average time taken to prepare meals (8). These consumers tend to substitute fresh potatoes for processed potato products to reduce the amount of time spend during cooking. Also, changes in food choices (5) and globalization of the food market (9) has shifted the demand for fresh potatoes toward processed ones.

Market data by Kantar Worldpanel shows that in 2017 the value sales for fresh potatoes declined by 2 per cent compared to the previous year. During the same period, the value sales for processed potatoes increased by 2.3 per cent (10). According to the (11), the downward demand for fresh potatoes could be attributed to the shift toward ready meals or convenience foods over the last decades.

According to Candel (12) and Darian and Cohen (13), convenience foods are products that help to reduce consumers time, physical and mental effort related to cooking activities. According to Devlin (14) the convenience retail food segment which includes ready-to-eat meals and soups contributed average revenue of about 6.2 billion pounds to the UK food retail market.

Despite the significant contribution of potato to UK food consumption, little has been done to understand consumer demand for processed and fresh potatoes. Such an analysis will help us understand to what extent demand for processed potatoes is influenced by the demand for fresh potatoes. And whether price changes in the potato market will have adverse consequences on average nutrient intake and the overall diet. As a result, the goal of this study is to estimate the demand for potatoes in the UK using data from Kantar Worldpanel 2018. Specifically, this study addresses the following research questions: (1) To what extent does price changes in the processed potato affect the demand fresh potatoes? (2) To what extent do different demographic groups respond to price changes in the fresh and processed potatoes market? Finally, what is the possible nutrient trade-off due to substitution effect between fresh and processed potatoes? These questions were addressed by estimating price and expenditure elasticities for eleven fresh potato products^2^, two processed products (convenience products) and a numeraire (encompassing all other food products).

## Conceptual Framework

### The LA/EASI Demand System

This study uses the Exact Affine Stone Index (EASI) demand system proposed by Lewbel and Pendakur (15) to estimate the demand for processed and unprocessed potato. The EASI demand system relate the budget share *w*_*i*_, to the polynomials of real food expenditure *y*_*i*_, vector of demographic characteristics *z*_*i*_, and vector of prices *p*_*i*_. The LA/EASI demand system budget share, *w*_*i*_ of each food is represented by:

(1) $$\begin{array}{l} w_{i}=\sum_{r=0}^{5}b_{r}y_{i}^{r}+Cz_{i}+Dz_{i}y_{i}+Ap_{i}+Bp_{i}y_{i}+\epsilon_{i} \end{array}$$

where *y*, real food expenditure is specified as:

(2) $$\begin{array}{l} y_{i}=\ln\left(x_{i}\right)-{p^{\prime }}_{i}w_{i} \end{array}$$

The variable *x*_*i*_ in (2) is the total household weekly expenditure, and matrices of parameters to be estimated are *A*, *B*, *C*, *D*, and *b*_*r*_.

Adding up and homogeneity of the cost function requires that the following restrictions should hold:

$$\begin{array}{r} {1^{\prime }}_{n}A={1^{\prime }}_{n}B={0^{\prime }}_{n};{1^{\prime }}_{n}C={1^{\prime }}_{n}D=0_{n}\\ {1^{\prime }}_{n}b_{0}=1,{1^{\prime }}_{n}b_{r}=0\;\forall r\neq 0 \end{array}$$

Symmetry of A and B Ensures Slutsky Symmetry.

The EASI demand system does not yield traditional Marshallian demand functions, rather implicit Marshallian demand equations. As a results, Marshallian demand elasticities are indirectly derived from the Hicksian price elasticities and expenditure elasticities using Slutsky equation (15).

Due to the high proportion of households reporting zero expenditure for most food groups, the consistent two-step estimation procedure by Shonkwiler and Yen (16) was applied to our system of censored equations. The estimation technique consists of two steps: (1) estimation of a probit equation and (2) the estimation of the EASI demand system (15).

The first step is a general probit mechanism, which includes thirteen equations, whether consuming Organic Potatoes and Vegetables, White Old Potatoes, Red Old Potatoes, Maris Piper Old Potatoes, King Edwards, Other Old Potatoes, New White Potatoes, Other New Potatoes, New Potatoes Baby, Baking Potatoes, Other Vegetables and Salads, Mashed Potatoes, and Frozen chips and other potatoes in the analysis. The probit equation for the *i*-th food group is represented as:

(3) $$\begin{array}{l} w_{i}^{*}=\;{X^{\prime }}_{i}\beta_{i}+\varepsilon_{i};\;d_{i}^{*}=\;{Z^{\prime }}_{i}v_{i}+\mu_{i} \end{array}$$

(4) $$\begin{array}{l} d_{i}=\left\{\begin{array}{c} 1\;if\;d_{i}>0\\ 0\;if\;d_{i}<0 \end{array}\right\}\;w_{i}=d_{i}w_{i}^{*} \end{array}$$

$w_{i}^{*}$ is the latent variable for the budget share, $d_{i}^{*}$ is the latent variable for the probit equation, *w*_*i*_ and *d*_*i*_ are the observed dependent variables, X_i_ and Z_i_ are vectors of exogenous variables determining level and participation, respectively, β_*i*_ and *v*_*i*_ are comfortable parameter vectors, ε_*i*_ and μ_*i*_ are error terms. Using the vector of parameter estimates, a set of CDF $\Phi \left(Z_{i}^{\;\prime }v_{i}\right)$ and PDF Φ $\left(Z_{i}^{\prime }v_{i}\right)\;$ are calculated and used in the second step to estimate the demand for potato.

### Price and Expenditure Endogeneity

Price and expenditure endogeneities are two main sources of inconsistency and biasedness in parameter estimates. LaFrance (17) and Lewbel and Pendakur (15) suggest existence of endogeneity in the demand system due to the correlation between error terms in each equation and the total real expenditures (*y*) (15, 17). To address this type of endogeneity, we first tested for endogeneity in our model by following the approached used by Blundell and Robin (18). We augmented each equation in the system (1) with the error term *v*_*i*_ from a reduced form expenditure equation (5).

(5) $$\begin{array}{l} \ln\left(X_{i}\right)=\alpha_{0}+\alpha_{2}.Region_{r}+\alpha_{2}.{\bar{X}}_{r}+\alpha_{3}.Time_{i}\\ \;+\sum_{i}^{n}\beta_{i}.\ln\left(Price\right)+v_{i} \end{array}$$

The reduced form of expenditure (*X*_*i*_) equation follows Blundell & Robin's specification and is defined as a function of log prices Price, regional dummies, mean household expenditure in region r (${\bar{X}}_{r}$), and number weeks the household bought potatoes (*Time*_*i*_). The hypothesis that the coefficient of the error term (*v*_*i*_) from the reduced parameters are different from zero was used to test the endogeneity of *X* (18, 19).

Similarly, to determine the presence of price endogeneity in our data, we regressed the endogenous variable (price) on a set of exogenous variables (demand and supply shifters) to generates residual errors (π_*i*_) [see (20)].

(6) $$\begin{array}{l} P_{i}=\beta_{0}+\beta_{1}.Prom_{i}+\beta_{2}.Share_{i}+\sum_{i}^{n}\beta_{i}.\ln\left(Price\right)+\pi_{i} \end{array}$$

Supply side factors include percentage of each product bought in each region (*Share*) and promotional indices (*Prom*). Demand shifters are log prices *ln(Price)* shown in Table 2. The residuals were included as independent variables in the EASI demand model (1) and tested for the significance of the corresponding parameter. A significant parameter suggests the presence of price endogeneity in the model. In the presence of endogeneity, the model (1) will be estimated by iterative three stage least squares using income groups as instrument.

### Data

This study relied on 2018 home scanner data purchased from Kantar Worldpanel (21). The Kantar Worldpanel data consist of household food purchases and demographic data. Each household that participated in the data collection process was given a scanner to scan the Universal Product Code (UPC) information of all products bought from retailers. The information retrieved from consumers include purchase store type, price and weight of the product, unit of measurement (i.e., grams, liters, or units), product-specific details (such as container type, barcode, and flavor). In addition, the home scan data contained information on household characteristics. Households' daily expenditures on both processed and unprocessed potato products were totalled to obtain weekly expenditures on the aggregated food groups.

A total of 19,726 households that hsad remained in the sample for at least 40 weeks were considered for our analysis. Based on the UK food and nutritional guidelines, 13 potato aggregates (11 fresh potato varieties and 2 processed potato products) and one numeraire were considered: (1) organic potatoes and vegetables, (2) white old potatoes, (3) red old potatoes, (4) Maris piper old potatoes, (5) king Edwards, (6) other old potatoes, (7) new white potatoes, (8) other new potatoes, (9) new potatoes baby, (10) baking potatoes, (11) other vegetables and salads, (12) prepared mashed potatoes^3^, (13) frozen chips and other potatoes and (14) miscellaneous foods.

### Summary Statistics

Table 1 reports the budget shares, promotional indices and the extent of censoring. Other Vegetables and Salads had the lowest censoring of about 0.97 per cent while Other New Potatoes had the highest level of censoring represented by about 98.8 per cent. Missing prices for non-consuming households were estimated using adjacent prices from neighboring cities in the UK. Among the fresh potato category, the most consumed is other vegetables and salads whilst the least consumed was other new potatoes. Similarly, among the processed potatoes, frozen chips and other potatoes are the most consumed.

**Table 1:** Commodity group composition and summary statistics.

**Food commodity groups**	**Mean budget shares (%)**	**Promotional indices**	**Level of censoring (%)**
Organic potatoes and vegetables	0.11	0.38	68.68
White old potatoes	0.15	0.80	51.13
Red old potatoes	0.07	1.04	77.30
Maris piper old potatoes	0.14	0.71	55.44
King edwards	0.03	0.54	85.30
Other old potatoes	0.01	0.54	96.84
New white potatoes	0.17	0.79	38.76
Other new potatoes	0.00	0.08	98.80
New potatoes baby	0.08	1.21	59.55
Baking potatoes	0.19	0.86	41.05
Other vegetables and salads	3.53	1.26	0.97
Mashed potatoes	0.08	0.88	75.74
Frozen chips and other potatoes	0.69	1.99	14.07
Miscellaneous Foods	94.73	0.38	0.00

*Source: Own computation, 2020*.

Demographic characteristics used for the probit equation (3), EASI demand system, and the reduced form of real expenditure equation are presented on Table 2. About 26 per cent of the respondents that participated in the data were males. Household heads who were married person represented a lower proportion of the data with a percentage representation of 6 per cent. The mean age of the household head was 52 years indicating an aged population. The average number of adults and kids in a family were 2.2 and 0.5, respectively. Households in the upper and upper-middle social class^4^ were largely represented with about 62.1 per cent. However, lower social class are the least represented with a per centage of 7.7 per cent. Total of 68 per cent of the households were found in the North of England (27 per cent), Midlands (17 per cent) and South of England (24 per cent). About 0.02 per cent of the respondents did not answer their employment status. Household heads who work over thirty hours a week represented the largest proportion with 38 per cent.

**Table 2:** Descriptive statistics of household composition and characteristics.

**Variable**	**Mean**	**Standard Error**
Gender (Male = 1)*	25.9%	0.003
Marital status (Married = 1)*^‡^	6.1%	0.002
Age*^‡^	52.034	0.099
Number of adults*^‡^	2.211	0.024
Number of kids*^‡^	0.547	0.007
**Social Class**		
Upper*	21.50%	0.003
Upper-Middle*	39.50%	0.003
Lower-Middle*	17.40%	0.003
Working*	13.80%	0.002
Lower*	7.70%	0.002
**Regions**		
Scotland^†^^‡^	9.00%	0.002
Wales^†^^‡^	5.00%	0.002
North^†^^‡^	27.00%	0.003
Midlands^†^^‡^	17.00%	0.003
East^†^^‡^	11.00%	0.002
South^†^^‡^	24.00%	0.003
London^†^^‡^	8.00%	0.002
**Employment**		
Over 30 h^†^	38.00%	0.003
8–29 h^†^	20.00%	0.003
Under 8 h^†^	2.00%	0.001
Unemployed^†^	2.00%	0.001
Retired^†^	25.00%	0.003
Full time education^†^	0.00%	0.000
Not working ^†^	12.00%	0.002
Unknown^†^	0.00%	0.000
**Income Ranges^↓^**		
0–£9,999 pa	5.4%	0.002
£10,000–£19,999 pa	19.0%	0.003
£20,000–£29,999 pa	18.6%	0.003
£30,000–£39,999 pa	14.3%	0.002
£40,000–£49,999 pa	10.2%	0.002
£50,000–£59,999 pa	6.5%	0.002
£60,000–£69,999 pa	3.8%	0.001
£70,000+ pa	5.4%	0.002
Unknown	16.7%	0.003

† Refers to demographic variables used in the reduced form expenditure equation; *Refers to demographic variables used in the EASI demand system;

‡ *Refers to demographic variables used in the probit equation*;

↓ *refers to instruments in the £3SLS*.

## Estimation Procedures

A standard Exact Affine Stone Index (LA/EASI) is used in the estimation. We modified the EASI incomplete demand system to account for censoring as follows:

(7) $$\begin{array}{l} w_{i}^{*}={\hat{\Phi }}_{i}\left(\sum_{r=0}^{5}b_{r}y_{i}^{r}+Cz_{i}+Dz_{i}y_{i}+Ap_{i}+Bp_{i}y_{i}\right)+\delta {\hat{\phi }}_{i}+\epsilon_{i} \end{array}$$

where ${\hat{\Phi }}_{i}$ and ${\hat{\phi }}_{i}$ are nxn identity matrices where the ones have been replaced by the cdf and pdf values, and δ is an *n* vector of parameters to be estimated. Economic theory does not provide any guidance regarding the selection of socio-demographic variables for the sample selection probit model (*x*_i_ vector) and those included in the demand equation (*z*_i_ vector). However, additional demographic variables are included in the *x*_*i*_ vector to avoid potential multicollinearity problems in the estimation of the censored model.

The final LA/EASI demand system taking into account zero purchases, price and expenditure endogeneity without interactions^5^ is represented as:

(8) $$\begin{array}{l} w_{i}={\hat{\Phi }}_{i}\left(\overset{5}{\underset{r=0}{\sum }}b_{r}y_{i}^{r}+Cz_{i}+Ap_{i}\right)+\delta {\hat{\phi }}_{i}+\rho v_{i}+\tau \pi_{i}+\epsilon_{i} \end{array}$$

The co-efficients, ρ* and τ* were tested for exogeneity of real food expenditure and prices.

The initial model was estimated using Iterative Seemingly Unrelated Regression (ISUR) estimator using all N equations. The estimation of all N equations was possible since the system of equations do not have a singular variance-covariance residual matrix [see (22)]. The coefficients ρ and π_*i*_ were tested for expenditure and price endogeneity, respectively. In the presence of endogeneity, iterative three stage least square (3SLS) was performed using income dummies as instruments to correct for the endogeneity.

### Estimating Elasticities

Expenditure elasticities, Hicksian and Marshallian price elasticities were derived from (8) following Castellón et al. (22). The compensated Hicksian price elasticity of demand for good *k* with respect to the price of the good *j* was derived by

(9) $$\begin{array}{l} \in \;=\;{\bar{w}}^{-1}\Phi \left(A\right)+\Omega \bar{w}-I \end{array}$$

where ∈ is an nxn matrix of compensated demand elasticities, *w* is an identity matrix where the ones have been replaced by the commodities' budget shares, Ω is an *n x n* matrix of ones and I is an identity matrix.

The expenditure elasticities ϑ were subsequently derived by

(10) $$\begin{array}{l} \vartheta ={\left(\bar{w}\right)}^{-1}{\left(I+\Phi bp\right)}^{-1}\Phi b+1_{n} \end{array}$$

where ϑ is the *J X 1* vector of estimated expenditure elasticities, *b* are the expenditure semi-elasticity coefficients, *p* is vector of mean prices and 1_*j*_ is a *J* × 1 vector of ones. The matrix of uncompensated Marshallian elasticities, ε, were derived from the Slutsky equation by

(11) $$\begin{array}{l} \varepsilon =\;\in -\;\bar{w}\vartheta \end{array}$$

### Nutritional Trade-Offs

Table 3 presents the average nutrient per 100 grams of each potato products. Frozen chips and other potatoes (74.3 kcal/100 grams) had the highest calories per 100 grams whilst Other old potatoes (10.10 kcal/100 grams). Mashed potatoes even though processed had moderate calories (42.94 kcal/100 g). Frozen chips and other potatoes had the highest content of calories (2.7 grams/100 grams) followed by mashed potatoes (1.53 grams/100 grams). However, Organic potatoes and vegetables had the highest content of sugar (1.32/100 grams). The high caloric and fat contents of our processed potato groups are important and have implications for sustainable diets.

**Table 3:** Average nutrient contents per 100 grams of potato product.

**Nutrients**	**Energy (kcal/100 g)**	**Protein (g/100 g)**	**Carbohydrate (g/100 g)**	**Sugar (g/100 g)**	**Fat (g/100 g)**	**Saturates (g/100 g)**	**Fiber (g/100 g)**	**Sodium (g/100 g)**
Organic potatoes and vegetables	17.69	0.88	2.79	1.32	0.28	0.06	0.80	0.01
White old potatoes	29.39	0.69	6.22	0.29	0.23	0.03	0.62	0.00
Red old potatoes	30.65	0.69	6.25	0.30	0.31	0.04	0.67	0.00
Maris piper old potatoes	39.85	0.91	8.32	0.36	0.36	0.04	0.79	0.00
King edwards	48.43	0.98	9.30	0.39	0.94	0.09	0.95	0.00
Other old potatoes	10.10	0.23	2.27	0.12	0.03	0.01	0.21	0.00
New white potatoes	24.98	0.59	5.57	0.32	0.08	0.02	0.54	0.00
Other new potatoes	45.40	1.04	9.84	0.66	0.12	0.04	0.82	0.00
New potatoes baby	28.19	0.67	6.21	0.41	0.09	0.02	0.60	0.00
Baking potatoes	41.01	1.02	9.11	0.47	0.08	0.02	0.92	0.00
Other vegetables and salads	18.18	0.85	2.34	1.25	0.62	0.11	0.90	0.02
Mashed potatoes	42.94	0.88	6.04	0.48	1.53	0.94	0.75	0.07
Frozen chips and other Potatoes	74.30	1.18	10.81	0.62	2.70	0.52	1.26	0.07

*Source: Own computation based on Kantar Worldpanel data*.

The nutritional trade-off between fresh and processed potatoes were analyzed from the context of change in demand using the estimated own price and cross-price elasticities from (13). A positive cross-price elasticity suggests that the two products are substitutes. We assumed that a fall in the price of processed potatoes due to promotion will have two effects: (1) fall in the quantity of fresh potatoes, (2) increase in the quantity of processed potatoes. Nutritional trade-offs were analyzed from the total effect of the price change i.e., sum of the substitution effect and the price effect. The nutritional trade-offs were estimated using the average weekly consumption (*Q*_*av*_), average weekly nutrient intakes (*q*_*av*_) and estimated cross-price elasticities (ε_*i,j*_).

The change in average weekly consumption was estimated using:

(12) $$\begin{array}{l} \Delta Q_{w}=\varepsilon_{i,j}\;*\;Q_{av} \end{array}$$

The change is weekly nutrient intake due to the 10 per cent price increase was estimated using:

(13) $$\begin{array}{l} \Delta q_{w}=\frac{Q_{w}}{Q_{w}}\;*\;q_{av} \end{array}$$

Results from the nutritional trade-offs are presented by bar charts in percentages.

## Results

We first tested for exogeneity of the expenditure and price variables in the data. The approach used to test for endogeneity was based on Blundell and Robin (18). Twelve out of fourteen coefficients of the residuals from the reduced form expenditure equation were significant, therefore, endogenous. Similarly, all the fourteen coefficients of the residual from the reduced form price equations were also endogenous. As a result, instead of estimating the demand system by iterative seemingly unrelated regression, iterative three stage least square (3SLS) was used to obtain consistent and efficient estimates.

### Expenditure Elasticities

**Figure 2:**
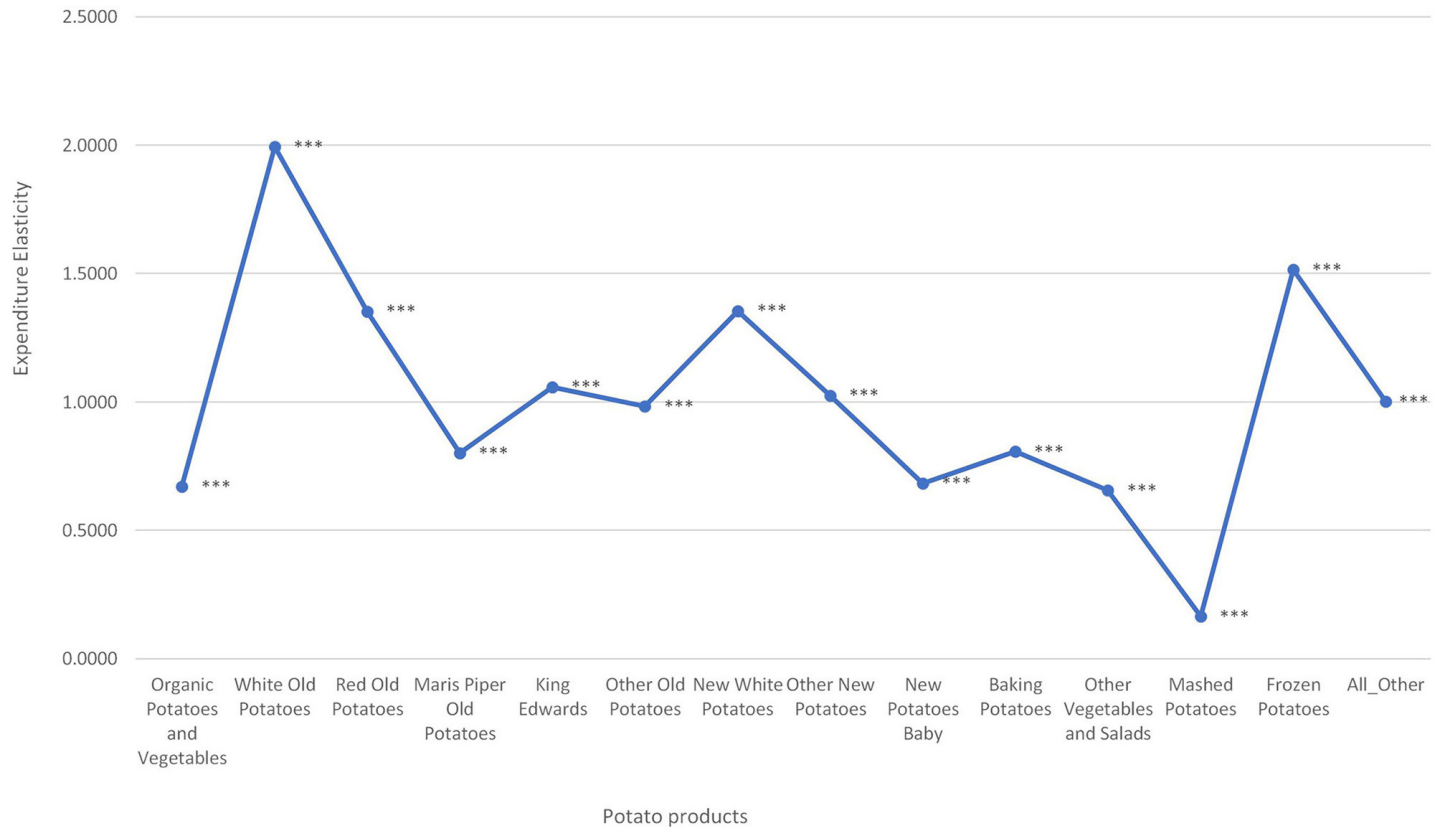
Average expenditure elasticity for potato types consumed in the UK. ***Indicates statistically significant at the 1% level.

First, Figure 2 shows the average household expenditure elasticity of thirteen different types of potatoes and miscellaneous foods consumed in the UK. Expenditure elasticities are between 0.17 (Mashed potatoes) and 2.00 (White old potatoes). Among the fresh potato types, other vegetables and salads are the least responsive to expenditure changes whilst white old potatoes are the most responsive to expenditure changes. Two types of processed potatoes were considered: Mashed and Frozen chips and other potatoes. Mashed potatoes were found to be less responsive to expenditure changes compared to frozen chips and other potatoes.

**Figure 3:**
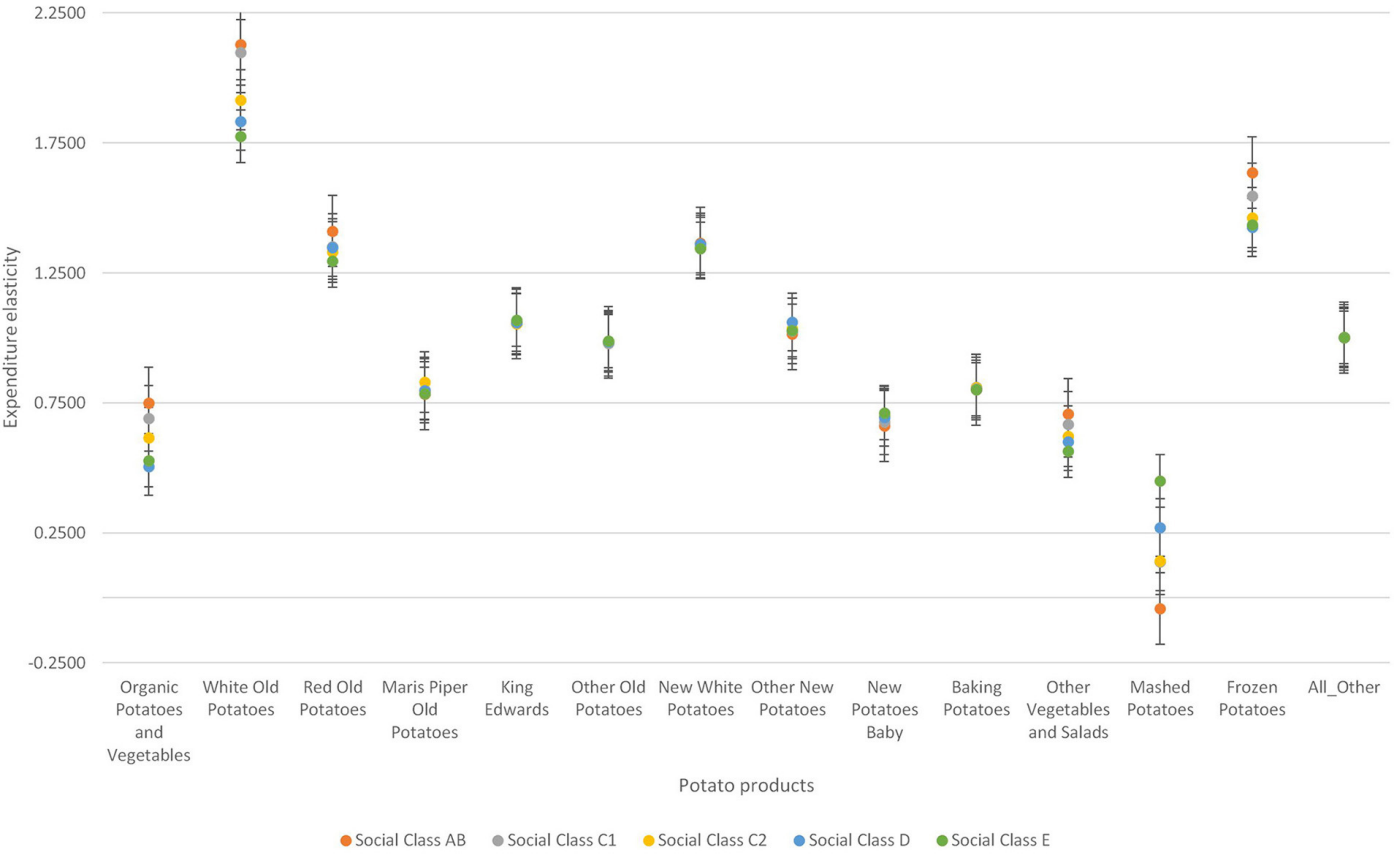
Variations in expenditure elasticities across different social class in the UK.

Second, the two demographic variables—social class and gender of the household head also influence demand for fresh and processed potatoes. Variations in the degree of responsiveness to expenditure changes across the different social class is prominent among mashed potatoes (see Figure 3). Among the fresh potatoes, variations in expenditure elasticities is prominent among organic potatoes and vegetables, white old potatoes, red old potatoes, and other vegetables and salads. Among the processed potatoes, consumers show high variation in the degree of responsiveness to expenditure changes in mashed potatoes category. Individuals within the upper social class are most responsive to expenditure changes in organic potatoes and vegetables, white old potatoes, red old potatoes and other vegetables and salads than any other social class. However, individuals classified as social working class are the least responsive to expenditure changes to organic potatoes and vegetables. Also, individuals classified as lower social class are the least responsive to expenditure changes in white old potatoes and red old potatoes. Households regarded as upper social class are the least responsive to expenditure changes in mashed potatoes but the most responsive to expenditure changes in frozen chips and other potatoes. The reverse is true for households in the lower social class.

**Figure 4:**
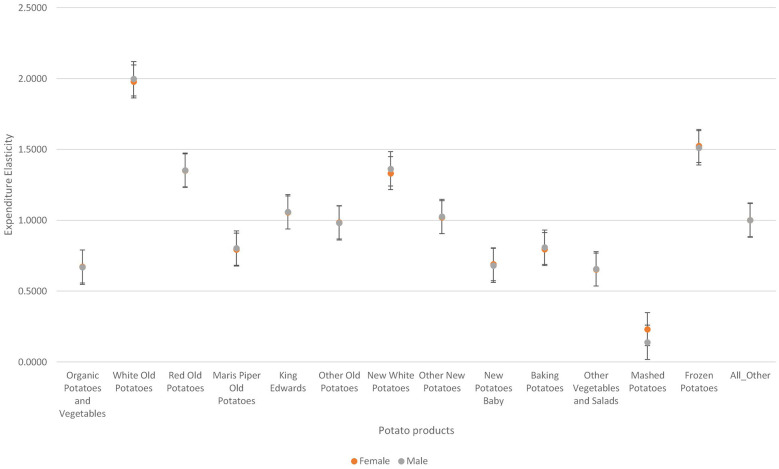
Variations in expenditure elasticities across sexes.

Figure 4 shows that there is little variation in the degree of responsiveness to expenditure changes across households based on the gender of the household head. Among the fresh potato types, variations are more pronounced for white old potatoes, new white potatoes and baking potatoes. On the other hand, among the processed potatoes, variations in responsiveness to price expenditure changes is more pronounced for mashed potatoes.

**Figure 5:**
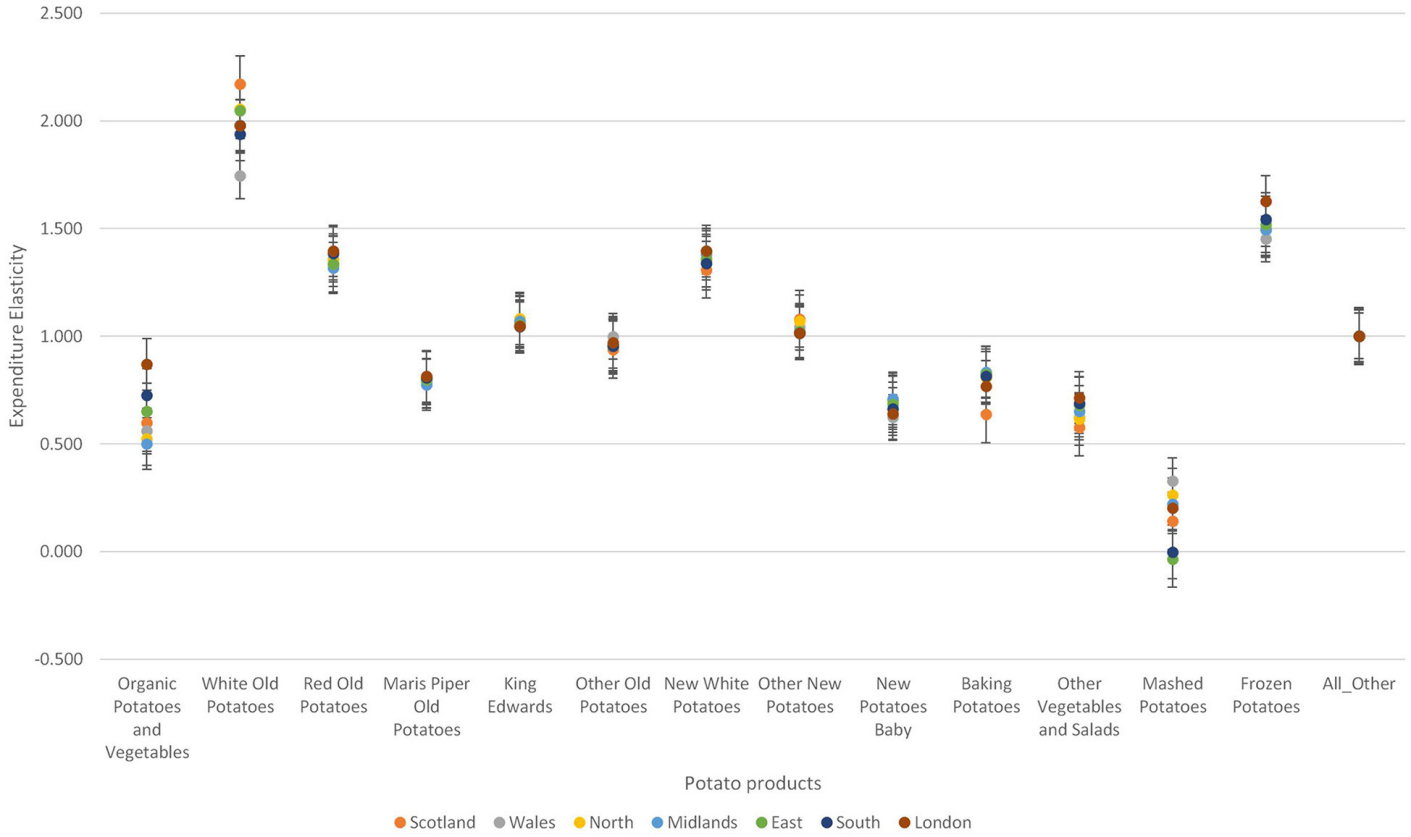
Variations in expenditure elasticities across regions in the United Kingdom.

Third, according to Figure 5 there are regional differences in the degree of responsiveness to price changes among the different potato types. For fresh potatoes, variations in the degree of responsiveness across the seven regions are more pronounced for organic potatoes and white old potatoes. Similarly, for processed potatoes, the variations in the degree of responsiveness to expenditures across the seven regions is more pronounced for mashed potatoes. Individuals living in London are the most responsive to expenditure changes in organic potatoes and vegetables, red old potatoes, Maris piper old potatoes, new white potatoes, baking potatoes, and other vegetables and salads. Scottish are more responsive to expenditure changes in white old potatoes; however, Scottish are the least responsive to expenditure changes in baking potatoes, and other vegetables and salads. For mashed potatoes, persons living in East London are the least responsive to expenditure changes whilst person living in Wales are the most responsive to expenditure changes. Persons living in London are more responsive to expenditure changes in frozen chips and other potatoes whilst persons living in Wales are the least responsive to expenditure changes in this food category.

The estimated average Marshallian price elasticities are reported in Table 4. All estimated own price elasticities are negative and significant. Among the fresh potatoes, demand for new white potatoes is the most price sensitive whilst the demand for other vegetables and salads is the least price sensitive. For the processed potatoes, demand for mashed potatoes is more price sensitive than frozen chips and other potatoes. New potatoes baby and baking potatoes were found to be substitute for Mashed potatoes. Maris piper old potatoes and new potatoes baby were also found to be substitute to frozen chips and other potatoes and white old potatoes and other vegetables and salads were complementary to frozen chips and other potatoes. The complementarity seems to suggest that buyer of potatoes usually purchase different types of potato for different purposes.

**Table 4:** Uncompensated demand elasticities for fresh and processed potatoes in the United Kingdom.

**Demands**	**Products**													
	**Organic potatoes and vegetables**	**White old potatoes**	**Red old potatoes**	**Maris Piper old potatoes**	**King Edwards**	**Other old potatoes**	**New white potatoes**	**Other new potatoes**	**New potatoes baby**	**Baking potatoes**	**Other vegetable and salads**	**Mashed potatoes**	**Frozen potato products**	**Other foods**
Organic potatoes and vegetables	−1.04^***^	0.14^**^	−0.04	0.05	−0.04	0.00^*^	0.08	0.00	−0.02	−0.08	0.74^***^	−0.01	−0.01	0.67^***^
White old potatoes	0.12^**^	−1.00^***^	0.04	0.09	0.04	0.00	0.08	0.00	−0.07	−0.02	−0.51^***^	0.02	−0.21^**^	1.22^***^
Red old potatoes	−0.03	0.03	−0.97^***^	−0.02	0.14^***^	−0.01^*^	0.32^***^	0.01^*^	0.11^***^	−0.11^**^	0.04	0.03	0.02	1.27^***^
Maris piper old potatoes	0.04	0.08	−0.02	−0.73^***^	0.11^***^	0.00	−0.06	0.00	0.09^*^	0.06	0.04	−0.02	0.16^***^	0.65^***^
King edwards	−0.07	0.07	0.30^***^	0.23^***^	−0.84^***^	−0.02	−0.24^***^	0.00	0.05	0.08	0.08	0.07	−0.02	1.01^***^
Other old potatoes	0.01^*^	−0.01	−0.03^*^	0.00	−0.02	−1.00^***^	0.00	0.00	0.02^***^	0.00	0.03^***^	0.00	0.01	0.93^***^
New white potatoes	0.07	0.09	0.36^***^	−0.07	−0.12^***^	0.00	−1.67^***^	0.01	0.35^***^	0.13^*^	0.01	0.07	−0.26	1.16
Other new potatoes	0.01	0.01	0.03^**^	0.00	0.01	−0.01	0.02	−1.01^***^	0.04^***^	0.02^**^	−1.63	0.00	−0.02	0.97^***^
New potatoes baby	−0.02	−0.13	0.21^***^	0.18^*^	0.05	0.02^***^	0.60^***^	0.02^***^	−1.63^***^	0.08	0.10	0.19^**^	0.26^***^	0.65^**^
Baking potatoes	−0.06	−0.02	−0.11^**^	0.05	0.03	0.00	0.11^*^	0.01^**^	0.04	−0.90^***^	−0.12	0.10^**^	0.00	0.85^***^
Other vegetables and salads	0.04^***^	−0.04^***^	0.00	0.00	0.00	0.00	0.00	0.00	0.00	−0.01	−0.57^***^	−0.01	−0.05^***^	0.60^***^
Mashed potatoes	−0.01	0.02	0.03	−0.02	0.03	0.00	0.07	0.00	0.11^**^	0.10^**^	−0.08	−1.35^***^	0.05	0.12^***^
Frozen chips and other potatoes	0.00	−0.07^**^	0.01	0.05	0.00	0.00	−0.08	0.00^***^	0.05^***^	0.00	−0.23^***^	0.01	−0.94^***^	1.24^***^
Other foods	0.00	0.00^***^	0.00^***^	0.00	0.00	0.00	0.00	0.00	0.00	0.00^***^	0.02^***^	0.00	0.01^***^	−0.05^***^

***, **, * *Indicates statistically significant at 1, 5, and 10%, respectively*.

**Figure 6:**
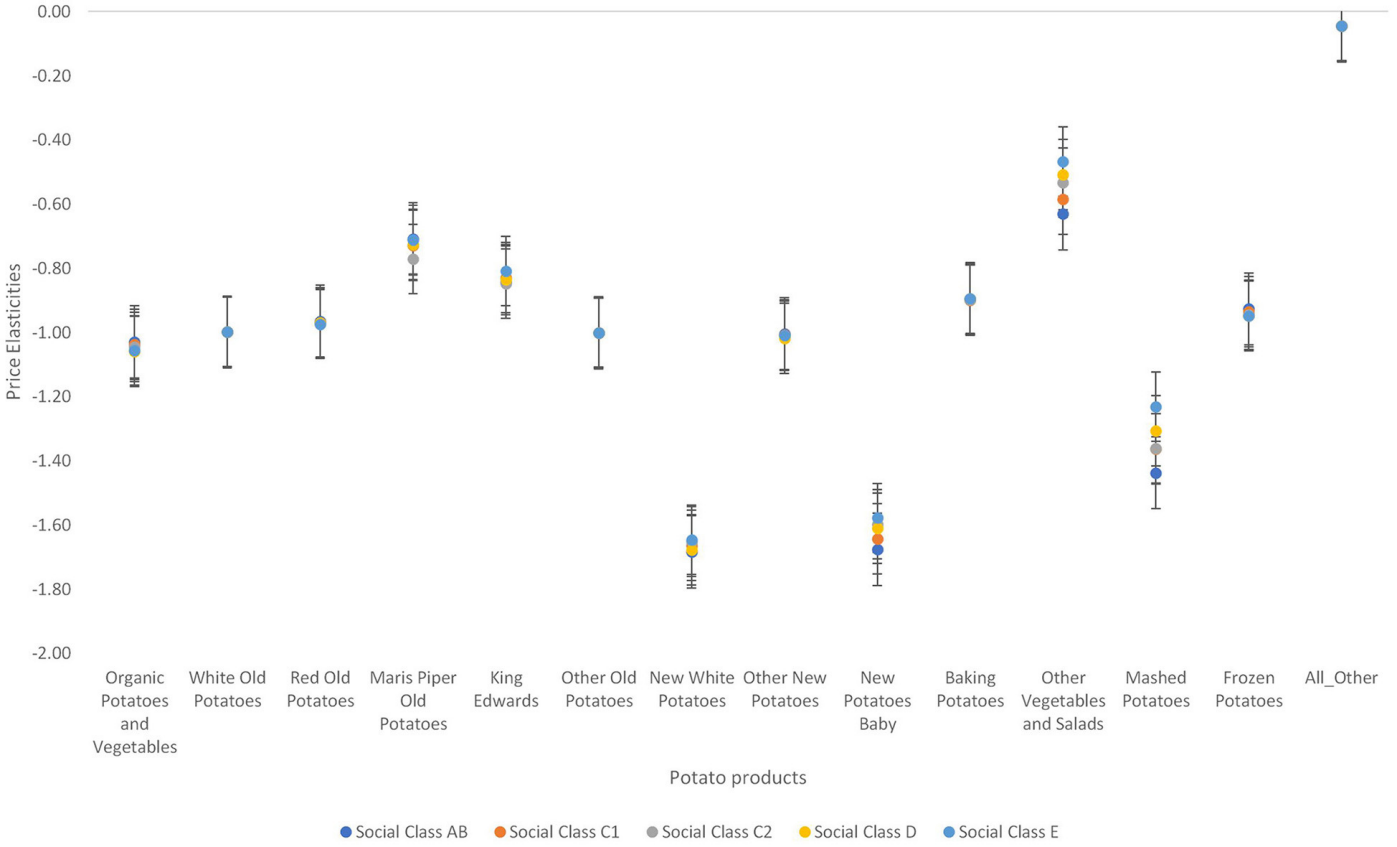
Variations in own price elasticities across different social class in the United Kingdom.

**Figure 7:**
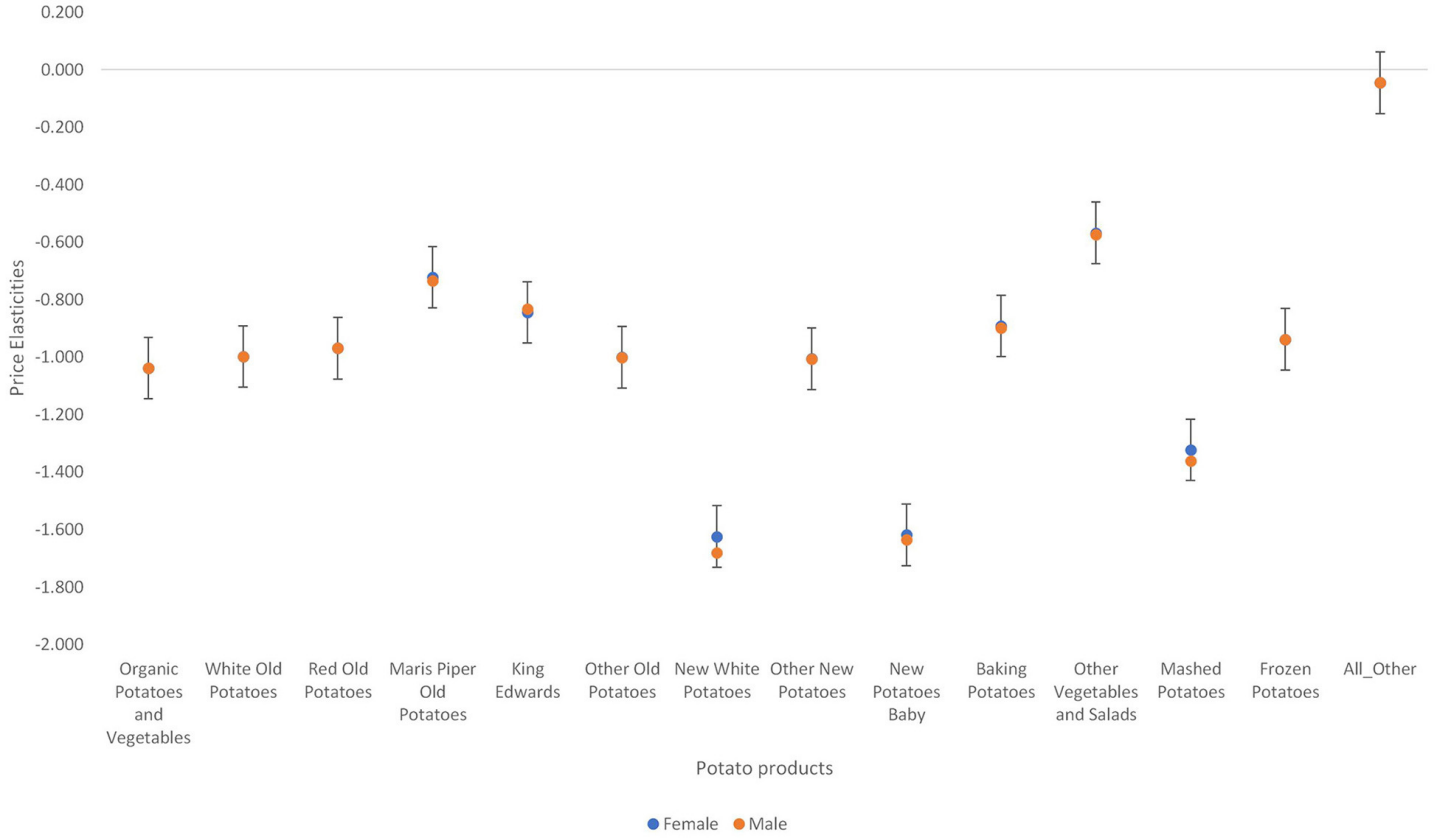
Variations in own price elasticities across sexes in the United Kingdom.

Second, Figures 6, 7 show there are demographic differences (in terms of sex and social class) in the price elasticities. Among the fresh potatoes, variations in price elasticities across the five social classes is more pronounced in Maris piper old potatoes, king Edwards, new white potatoes, new potatoes baby, and other vegetables and salads. Upper social classes are the most responsive to price changes in all these types of potatoes.

The variations in the price elasticities among females and males were not very pronounced (see Figure 7). New white potatoes and mashed potatoes were the two potato types that showed quite a significant variation. The pattern and degree of elasticity is the same as that of the average household. Females are more responsive to price changes in mashed potatoes and new white potatoes than males.

**Figure 8:**
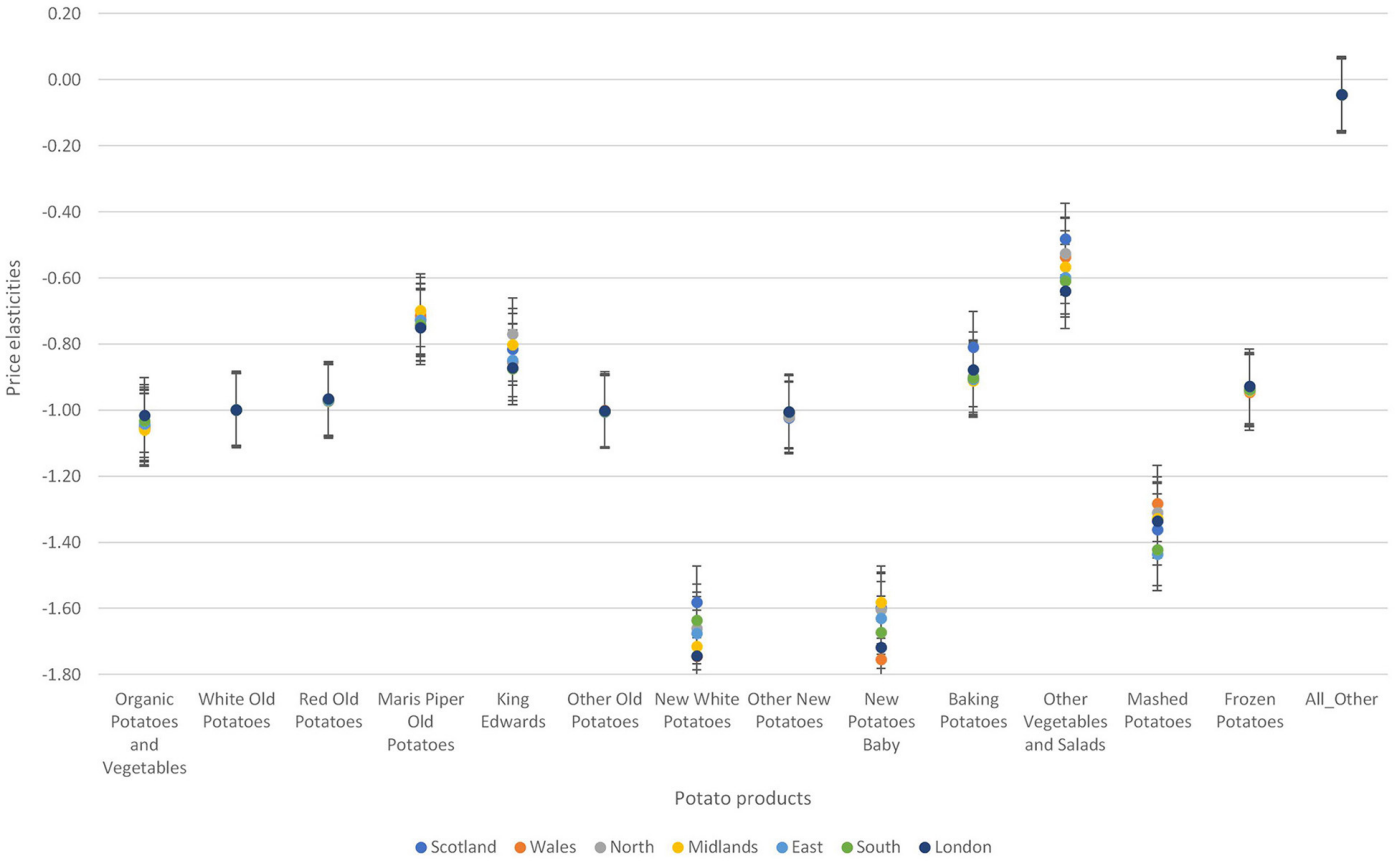
Variations in own price elasticities across different regions in the United Kingdom.

Third, there were regional differences in the degree of responsiveness to price changes across the seven regions in the UK (see Figure 8). Variations in the degree of responsiveness to prices are more pronounced for organic potatoes and vegetables, new white potatoes, baking potatoes, other vegetables and salad, and mashed potatoes. Individuals living in London are the least responsive to price changes in organic potatoes and vegetables, red potatoes, other old potatoes, and other new potatoes but most price responsive to price changes in Maris piper old potatoes, new white potatoes and new potatoes baby. The midlands are the least responsive to price changes in Maris piper old potatoes and new potatoes baby. For the processed potatoes, persons living in Wales are the most responsive to price changes in mashed potatoes and Person living in East London are the least responsive to price changes. Similarly, persons living in London are more responsive to price changes in frozen chips and other potatoes and persons living in Wales are the least responsive to price changes in frozen chips and other potatoes.

## Nutritional Trade-Offs: Fresh vs. Processed Potatoes

### Nutritional Trade-Offs From Substituting Fresh New Potatoes Baby for Mashed Potatoes

**Figure 9:**
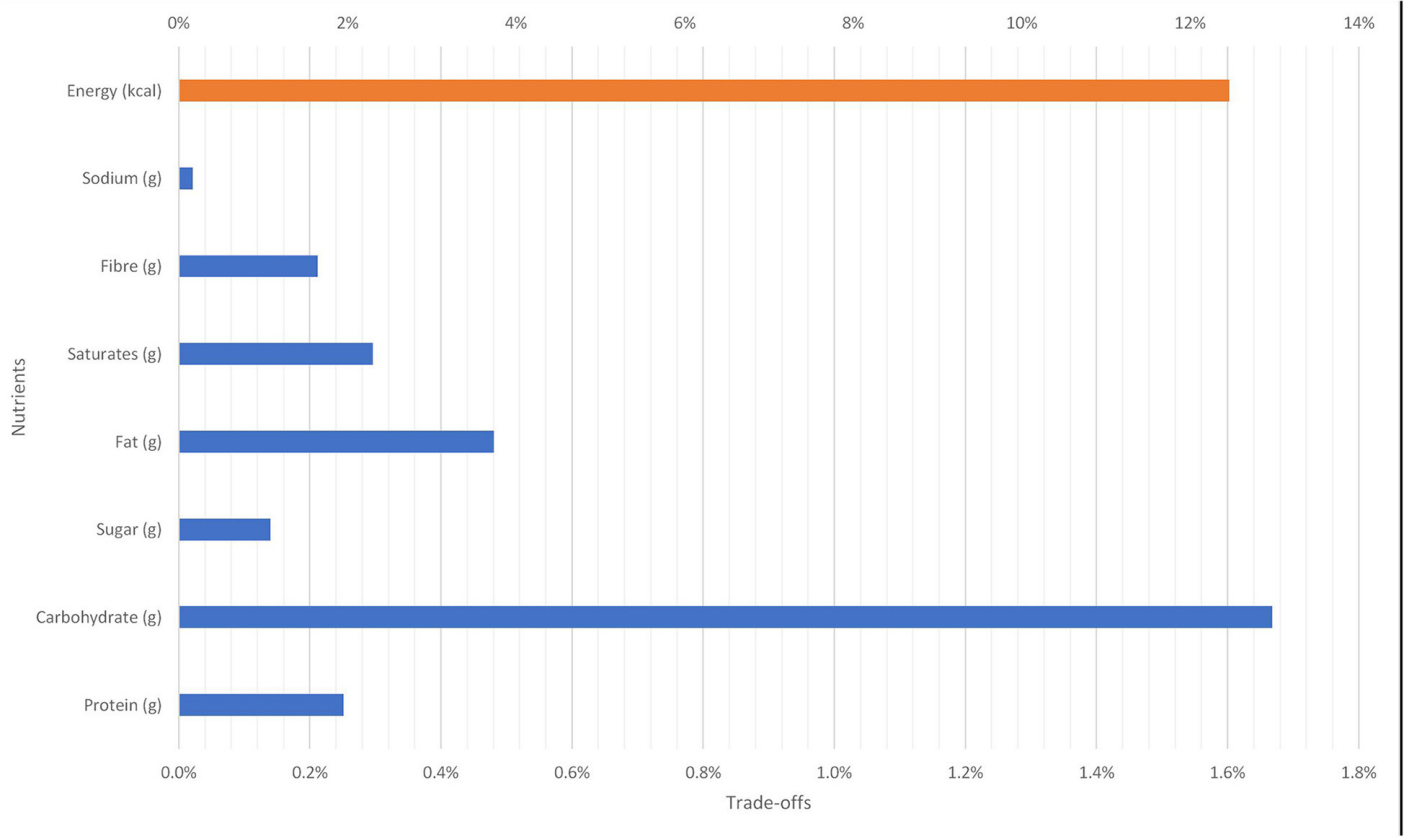
Nutritional trade-off due to 10 per cent reduction in the price of mashed potatoes on new potatoes baby.

Table 4 above shows a significant substitution between mashed potatoes and fresh potatoes. Since both products are substitutes, a price reduction in mashed potatoes due to promotions will have two effects; a decrease in the consumption of fresh potatoes and an increase in the consumption of processed potatoes. These changes have implications for weekly nutrient intake. Figure 9 shows the effect of a 10 per cent price reduction in mashed potatoes weekly nutrient intake.

On average, a 10 per cent reduction in the price of mashed potatoes will increase total caloric intake by about 12.4 per cent. In addition, there will be an increase in macronutrient intakes: carbohydrate (1.7 per cent), protein (0.25 per cent), and fats (0.48 per cent). The intakes of nutrients like sodium and unsaturated fat will also increase.

### Nutritional Trade-Offs From Substituting Fresh Baking Potatoes for Mashed Potatoes

**Figure 10:**
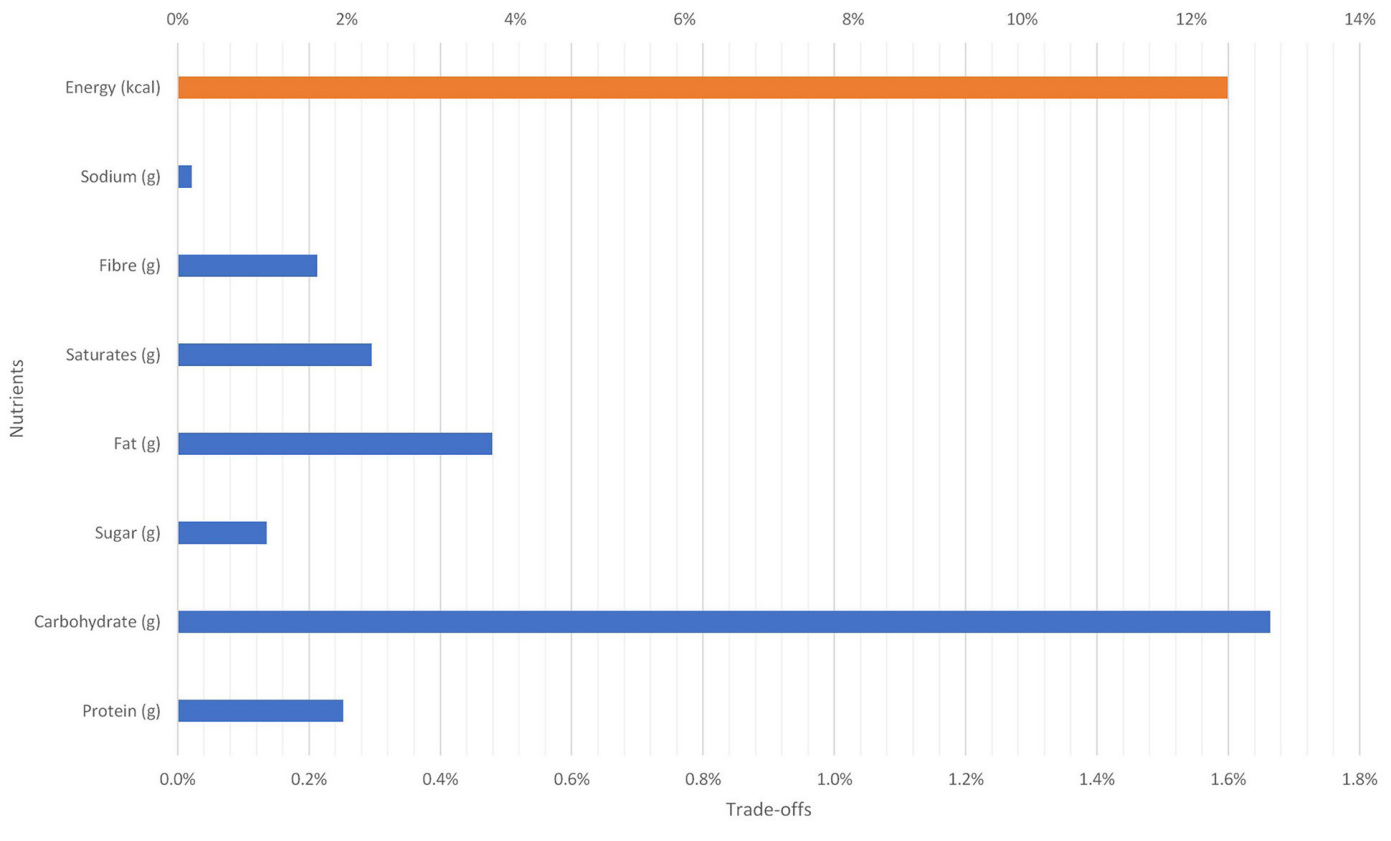
Nutritional trade-off due to 10% reduction in the price of mashed potatoes on baking potatoes.

Table 4 shows a significant cross-price elasticity between mashed potatoes and fresh new potatoes baby. Assuming a 10 per cent reduction in the price of mashed potatoes has implication for the consumption of fresh potatoes as well as mashed potatoes. The total effect of the price fall is shown in terms of nutritional trade-offs in Figure 10. Holding all other prices constant, it is expected that weekly caloric intake will increase by 12.5 per cent. In addition, the weekly average intakes of carbohydrate, protein, fats will increase by 1.7 per cent, 0.3 per cent, 0.5 per cent, respectively (see Figure 10). There will also be an increase in the intake of saturated fats and sodium.

### Nutritional Trade-Offs From Substituting New Potatoes Baby for Frozen Chips and Other Potatoes

**Figure 11:**
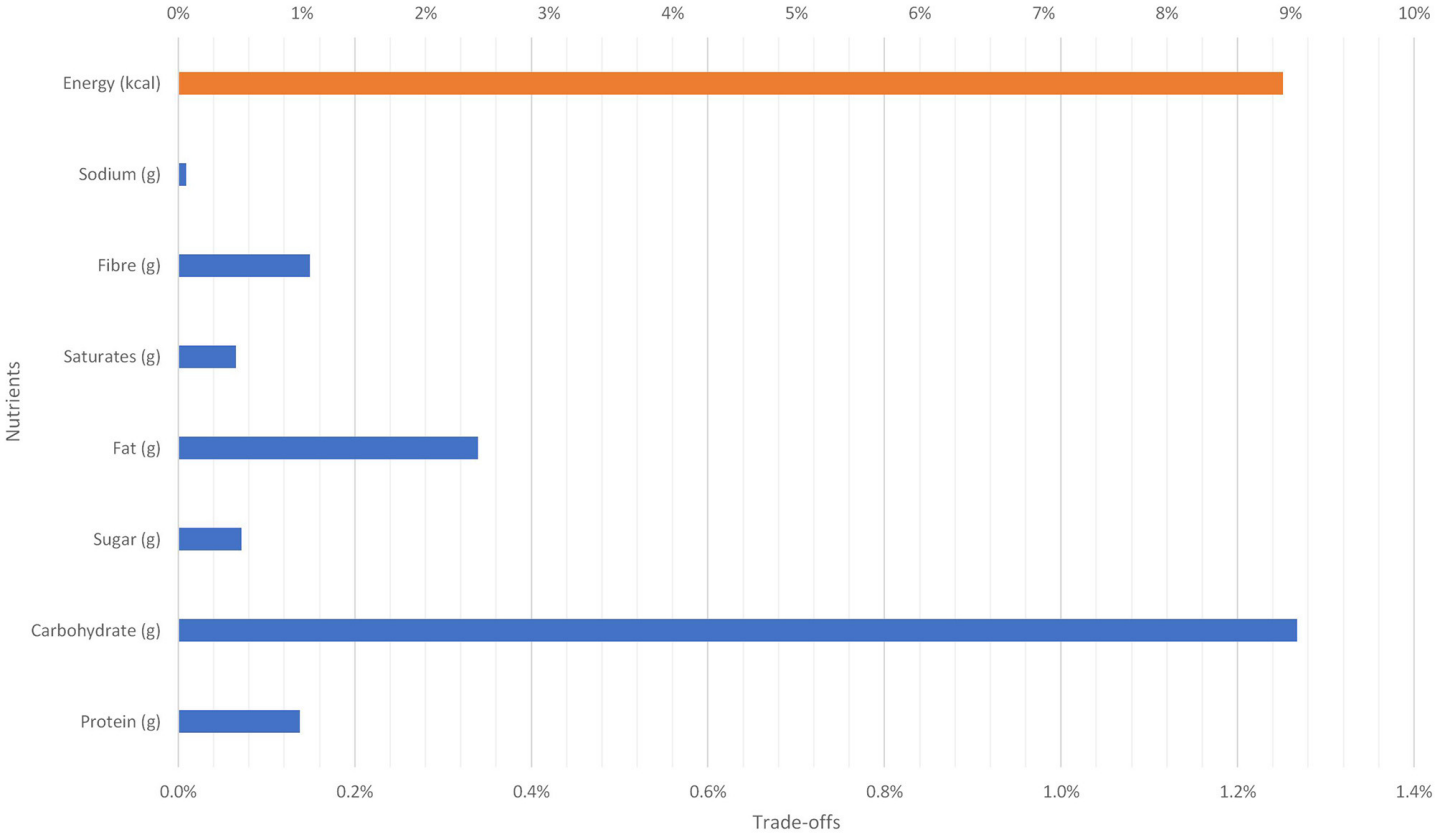
Nutritional trade-off due to 10% reduction in the price of frozen chips and other processed potatoes on new potatoes baby.

There was a significant positive relationship between new potatoes baby and mashed potatoes which has implications for retailers' pricing decisions. A pricing policy that makes frozen chips and other processed potatoes 10 per cent cheaper than fresh potatoes will increase the consumption frozen chips and other processed potatoes. The total effect on weekly nutrient intakes are shown in Figure 11. First, total weekly caloric intake will increase by 8.9 per cent. In terms of macronutrient intake, carbohydrate, protein and fats will increase by 1.3 per cent, 0.1 per cent, 0.3 per cent, respectively.

**Figure 12:**
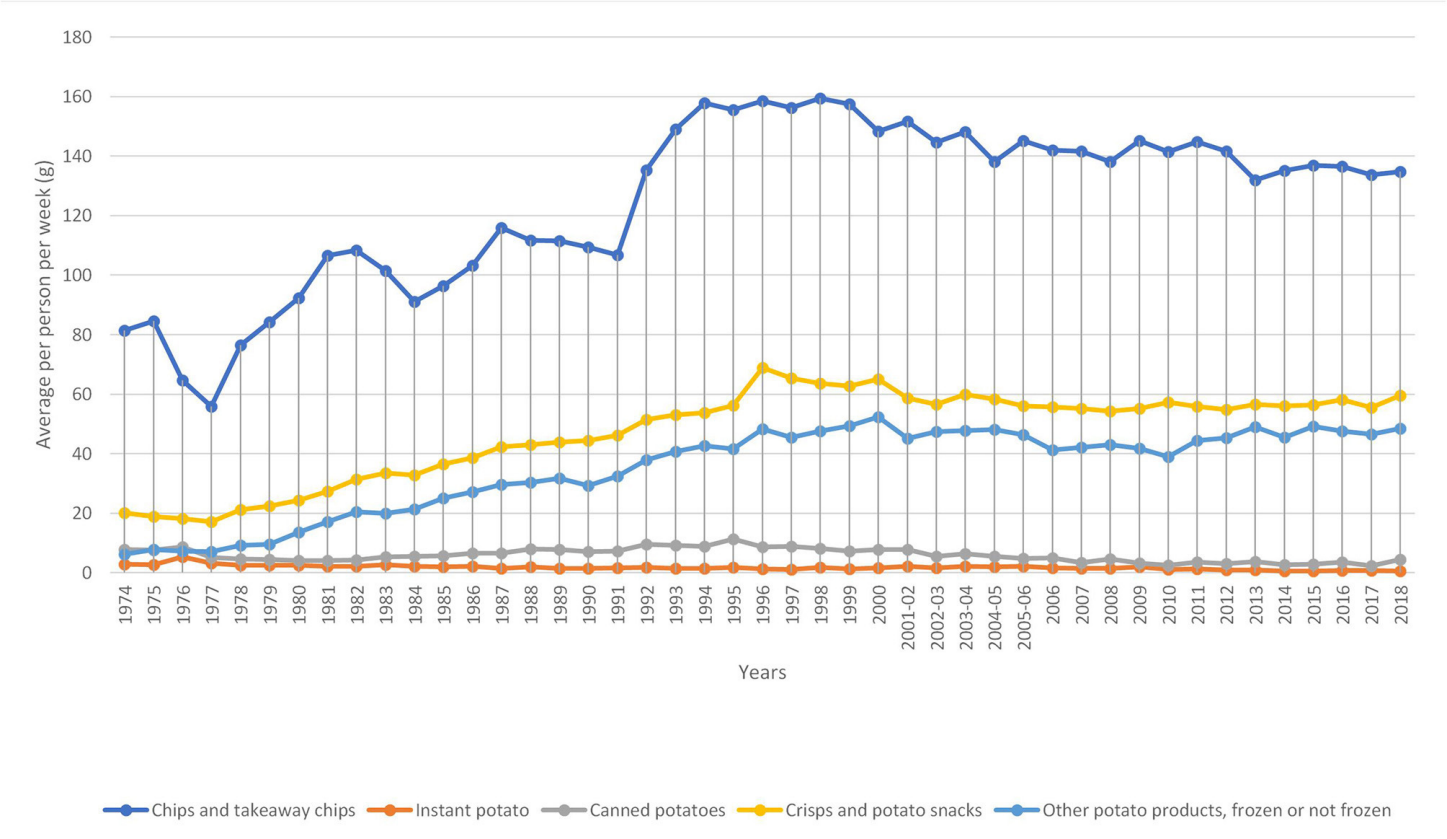
Average weekly consumption of different types of processed potato products in the UK. Source: Own elaboration based on DEFRA data.

Both processed and fresh foods contribute to food and nutrition security (23). However, increased consumption of processed foods has adverse effect on health (24, 25). Figures 9–12 show the implications of trading fresh potatoes for processed potatoes. Pricing policies that make processed potato products cheaper than unprocessed ones has implication for consumption and health. First, it is evident that total caloric intake will increase. Since current average UK caloric intake is above the recommended level (26), any policy that increases daily caloric intake is inappropriate. Second, the increase in the consumption of saturated fats and sodium has implications for the prevalence of obesity and associated diseases in UK. Finally, even though, the intake of protein increases, total dietary fat increases more offsetting the positive impact of the price fall.

### Implications of Nutritional Trade-Offs for Sustainable Dietary Goals

There is a conscious effort among policy makers and health professional to create more sustainable diets. It is therefore important to analyse how various groups of consumer derived their calories as well as dietary fat. Potatoes is a staple food in the UK and a major source of dietary calories, fat, and Vitamin C (5). According to the British Nutrition Foundation average caloric intake in the UK is on the decline, however the entire UK population more sedentary and obesity levels are on the increase. To maintain healthy weights, energy intake and energy expenditure should be balanced (27). As a result, any policy that destabilizes this necessary condition is not sustainable. For instance, Doubly-labeled water (DLW) studies of energy expenditure suggest that increased obesity prevalence reflects excessive food energy intake with physical activity levels unchanged (28). The present support findings that show that pricing policies that make processed potatoes cheaper do not promote sustainable consumption as the overall energy balance is destabilized. In effect, increase the average weekly caloric and fat intakes have consequences health goals targeted at obesity and non-communicable diseases (29). According the data by Scottish Government in 2010, if these trends continue the cost of obesity to the Scottish government will more than double by 2030 ranging from £0.9 billion—£3 billion (27).

## Final Remarks

The consumption of both processed and unprocessed foods is important for nutrition. There are no direct subsidies on processed foods like fries and chips or mashed potatoes, however, retailers' promotional activities make those foods cheaper relative to fresh products. We have shown in this paper that any pricing policy that lower the price of processed potato products relative to unprocessed potato products has three implications: (1) a reduction in the consumption of fresh potato products due to substitution effect; (2) an increase in the consumption of the relatively cheaper product, in this case, processed potato products; and (3) increase in average weekly caloric intake as well as increase in saturated fat and sodium.

These results have implications for public health and diet quality. First, substituting fresh potato products for processed potato products reduces the quality of consumers diet. This is evident from the increased intake of sodium and saturated fats. Moreover, in the UK, where per capita calories are above the recommended intake levels, any pricing policies that increases per capita caloric intake is detrimental to health and deserves a second look.

Second, high intakes of sodium and saturated fats are the major causes of cardiovascular diseases (CVDs) and certain cancers. As increases in the intake of both nutrients is likely to accelerate the prevalence of CVDs and cancers.

Finally, there is the need for future research to go a step further to estimate the impact of these dietary changes on Disability-adjusted life year (DALY) in the UK.

## Data Availability Statement

The data analyzed in this study is subject to the following licenses/restrictions: The authors does not have the rights to make the data public. Requests to access these datasets should be directed to Wisdom Dogbe, wisdom.dogbe@abdn.ac.uk.

## Ethics Statement

Ethical approval was not provided for this study on human participants because the data used in this research was secondary data (home scan panel data). The researchers had no influence on the manner in which the data was collected.

## Author Contributions

WD performed the literature review, data analysis and results. CR-G organized the data for the analysis and supervised the manuscript. Both authors contributed to the article and approve the submitted version.

## Conflict of Interest

The authors declare that the research was conducted in the absence of any commercial or financial relationships that could be construed as a potential conflict of interest.

## Footnotes

^1^Rate was calculated using growth rate model i.e. ln(Quantity) = a + b.Time.

^2^The use of fresh potatoes refers to all table stock potatoes whilst processed potatoes refers to potato products that have undergone some form of industry transformation.

^3^Mashed potato is a side dish, made from mashing boiled potatoes.

^4^Social class is defined by occupation, wealth and education. Upper Class - Higher managerial, administrative and Intermediate managerial, administrative or professional; Upper-Middle Class - Supervisory or clerical and junior managerial, administrative or professional; Lower-Middle Class - skilled manual workers; Working Class - Semi and unskilled manual workers; and Lower Class - Casual or lowest grade workers, pensioners and others who depend on the state for their income.

^5^Results from model with interactions and without interactions do not differ significantly.

## References

[1] Camire ME Kubow S Donnelly DJ. Potatoes and human health. Crit. Rev. Food Sci. Nutrit. (2009) 49:823–40. 10.1080/10408390903041996 19960391

[2] Gagnon R Drouin M Peters D Canadian potato situation and trends 2006–2007. Agricult Agri-Food Canada. (2007). p. 5–20.

[3] Struik PC Book Review: World Catalogue of Potato Varieties 2007, Hils U, and L. Pieterse (eds). AgriMedia GmbH, Clenze, Germany and Allentown, PA. USA. ISBN 978-3-86037-310-1. Published 2007. Price: Euro 98.00, USD 107.00. Potato Res. (2008) 51:209–10. 10.1007/s11540-008-9101-6

[4] FAO Statistical Department Food and Agricultural Data (2020). Available online at: http://www.fao.org/faostat/en/#home

[5] Riley H Potato consumption in the UK-why is' meat and two veg'no longer the traditional British meal? Nutrit Bull. (2010) 35:320–31. 10.1111/j.1467-3010.2010.01864.x

[6] Joint SACN/RCPCH Expert Group on Growth Standards GB Application of WHO Growth Standards in the UK. Stationery Office Books (TSO) (2008).

[7] Department of Environment Food and Rural Affairs (2020) (DEFRA) Family Food Datasets. GOV.UK. Available online at: https://www.gov.uk/government/statistical-data-sets/family-food-datasets

[8] Foster R Lunn J 40th Anniversary Briefing Paper: Food availability and our changing diet. Nutrit Bull. (2007) 32:187–249. 10.1111/j.1467-3010.2007.00648.x

[9] Leung G News and views: ethnic foods in the UK. Nutrit Bull. (2010) 35:226–34. 10.1111/j.1467-3010.2010.01840.x

[10] Agriculture and Horticulture Development Board (AHDB) (2017) Great Britain Poatoes Market 2016–2017. Available online at: potatoes.ahdb.org.uk

[11] Gregory J Lowe S Bates CJ. National Diet and Nutrition Survey: Young People Aged 4-18 Years, Vol. 1. Report of the Diet and Nutrition Survey. London: The Stationery Office (2000).

[12] Candel MJJM. Consumers' convenience orientation towards meal preparation: conceptualization and measurement. Appetite. (2001) 36:15–28. 10.1006/appe.2000.0364 11161342

[13] Darian JC Cohen J Segmenting by consumer time shortage. J Consumer Market. (1995) 12:32–44. 10.1108/07363769510146787

[14] Devlin E Food-to-go market set for growth in 2020 despite sector slowdown. The Grocer. (2020). Available online at: https://www.thegrocer.co.uk/convenience/food-to-go-market-set-for-growth-in-2020-despite-sector-slowdown/601763.article.

[15] Lewbel A Pendakur K Tricks with Hicks: the EASI demand system. Am Economic Rev. (2009) 99:827–63. 10.1257/aer.99.3.827

[16] Shonkwiler JS Yen ST Two-step estimation of a censored system of equations. Am J Agricult Econom. (1999) 81:972–82. 10.2307/1244339

[17] LaFrance JT When Is Expenditure “exogenous” in Separable Demand Models? Western Journal of Agricultural Economics (1991). p. 49–62.

[18] Blundell R Robin J.-M Latent separability: grouping goods without weak separability. Econometrica. (2000) 68:53–84. 10.1111/1468-0262.00093

[19] Boonsaeng T Fletcher SM Carpio CE European union import demand for in-shell peanuts. J Agricult Appl Econom. (2008) 40:941–51. 10.1017/S1074070800002431

[20] Dhar T Chavas JP Gould BW An empirical assessment of endogeneity issues in demand analysis for differentiated products. Am J Agricult Econom. (2003) 85:605–17. 10.1111/1467-8276.00459

[21] Kantar W SRUC Proprietary Database. (2018). Available online at: https://www.kantarworldpanel.com/global.

[22] Castellón CE Boonsaeng T Carpio CE Demand system estimation in the absence of price data: an application of Stone-Lewbel price indices. Appl Econom. (2015) 47:553–68. 10.1080/00036846.2014.975332

[23] Weaver CM Dwyer J Fulgoni VL King JC Leveille GA MacDonald RS. Processed foods: contributions to nutrition. Am J Clin Nutrit. (2014) 99:1525–42. 10.3945/ajcn.114.089284 24760975 PMC6410904

[24] Briggs M Petersen K Kris-Etherton P. Saturated fatty acids and cardiovascular disease: replacements for saturated fat to reduce cardiovascular risk. Healthcare. (2017) 5:29. 10.3390/healthcare5020029 28635680 PMC5492032

[25] Hall KD Ayuketah A Brychta R Cai H Cassimatis T Chen KY Las dietas ultraprocesadas provocan una ingesta excesiva de calorías y un aumento de peso: un ensayo controlado aleatorio para pacientes hospitalizados de la ingesta de alimentos ad libitum. Cell Metabolism. (2019) 30:67–77. 10.1016/j.cmet.2019.05.008 31105044 PMC7946062

[26] Pasha-Robinson L Britons Eating 50% More Calories Than They Realise, Reveals Study. The Independent (2018). Available online at: https://www.independent.co.uk/news/uk/home-news/uk-calories-eat-think-obesity-overweight-ons-study-british-a8217486.html.

[27] Scottish Government T Preventing Overweight and Obesity in Scotland: A Route Map Towards Healthy Weight. (2010). Available online at: www.scotland.gov.uk.

[28] Millward DJ Energy balance and obesity: a UK perspective on the gluttony v. sloth debate. Nutrit Res Rev. (2013) 26:89–109. 10.1017/S095442241300005X 23750809

[29] Fair AM Montgomery K. Energy balance, physical activity, cancer risk. In: Cancer Epidemiology, Methods in Molecular Biology Series, Vol. 472. Springer (2009). p. 57–88. 10.1007/978-1-60327-492-0_3 19107429

